# Invasive salivary duct carcinoma ex pleomorphic adenoma of the parotid gland: a teaching case giving rise to the genuine diagnostic difficulty on an inadequate cytology specimen

**DOI:** 10.1186/1746-1596-7-61

**Published:** 2012-05-30

**Authors:** Sohsuke Yamada, Atsunori Nabeshima, Takahisa Tabata, Xin Guo, Takashi Tasaki, Ke-Yong Wang, Shohei Shimajiri, Yasuyuki Sasaguri

**Affiliations:** 1 Departments of Pathology and Cell Biology School of Medicine, University of Occupational and Environmental Health, Kitakyushu, Japan; 2 Departments of Otorhinolaryngology, School of Medicine, University of Occupational and Environmental Health, Kitakyushu, Japan; 3 Department of Pathology and Cell Biology, School of Medicine, University of Occupational and Environmental Health, 1-1 Iseigaoka, Yahatanishi-ku, Kitakyushu, 807-8555, Japan

**Keywords:** Salivary duct carcinoma, Carcinoma ex pleomorphic adenoma, Salivary gland, Cytology

## Abstract

**Abstract:**

A history of a recent rapid increase in long-standing swelling mass was presented in the right parotid gland of an 85-year-old male. The inadequate cytologic specimens contained few small clusters of three-dimensional malignant epithelial cells having hyperchromatic pleomorphic nuclei and prominent nucleoli, adjacent to a cluster of benign monomorphic myoepithelial cells. We first interpreted it merely as an adenocarcinoma, not otherwise specified. A radical parotidectomy was performed, and gross examination revealed an encapsulated and firm tumor lesion, looking grayish-blue to yellowish-white, focally associated with extracapsular invasion. On microscopic examination, the tumor was predominantly composed of a proliferation of highly atypical epithelial cells having abundant eosinophilic cytoplasm, often arranged in a Roman-bridge appearance with foci of comedo necrosis, alternating with extensive infiltration to adjacent stroma in a trabecular or alveolar fashion with severe vessel permeation. Within the background of pleomorphic adenoma, the carcinoma cells sometimes replaced ductal luminal cells while retaining an intact-like myoepithelial layer. Therefore, we finally made a diagnosis of invasive salivary duct carcinoma ex pleomorphic adenoma. We should be aware that owing to its characteristic features, cytopathologists might be able to determine correct diagnosis, based on multiple and adequate samplings.

**Virtual slides:**

The virtual slide(s) for this article can be found here: http://www.diagnosticpathology.diagnomx.eu/vs/2126158270695815

## Background

Among all salivary gland neoplasms, carcinoma ex pleomorphic adenoma (Ca ex PA) accounts for approximately 3.6% [1], whereas constitutes 6.2% of all PA and 11.6% of all malignant salivary gland neoplasms [1,2]. Despite of that, Ca ex PA is uncommon and has a prevalence rate of 5.6 cases per 100,000 malignant neoplasms and a yearly incidence rate of 0.17 tumors per 1 million people [1,2]. This neoplasm is defined as an epithelial malignant transformation within a primary (de novo) or recurrent PA, and often poses a diagnostic challenge to clinicians and cytopathologists, since its entity is difficult to diagnose pre-operatively [3,4]. In fact, Ca ex PA can be asymptomatic as most of them are not widely invasive on gross findings and often have similar clinical presentations as PA, however, patients with Ca ex PA have a poor prognosis due to infiltrative and destructive behavior, and thus, early and accurate diagnosis and aggressive surgical treatment (i.e., total or radical parotidectomy) can increase their survival rates [1-5]. Although any form of carcinoma can be observed and also be a mixture of subtypes, the malignant component of Ca ex PA is most often adenocarcinoma, not otherwise specified (NOS), and sometimes, may be salivary duct carcinoma (SDC), undifferentiated carcinoma, adenoid cystic carcinoma, or mucoepidermoid carcinoma [1,2,4,6]. While, WHO states that its component is most frequently a poorly differentiated carcinoma, e.g., SDC or adenocarcinoma, NOS, or an undifferentiated carcinoma [3].

On the other hand, SDC was first described by Kleinsasser *et al* in 1968 [7], and to date, more than 100 cases have been reported and account for approximately 9% among all salivary gland neoplasms [8]. SDC is a distinctive but relatively common high grade adenocarcinoma arising from the excretory ductal epithelium of the major salivary glands, especially the parotid gland [8-10]. Clinically, these tumors are characterized by aggressive (i.e., infiltrative and destructive) behavior with local recurrence, early and distant metastasis, invasion of the facial nerve, and/or significant mortality [8-11]. Histopathologically, SDCs have a striking resemblance to ductal carcinoma of the breast, exhibiting intraductal and infiltrating components [8,10,11]. In addition, very intriguingly, they should show a broader clinicopathological spectrum and many cases of them may develop within PA as a result of malignant transformation of ductal epithelial cells [9,12]. It has been actually reported that multifocal origin of SDC from major excretory ducts surrounding a PA was found [8]. By contrast, Simpson RHW *et al.* described that the majority of them arise *de novo* (as in the breast) probably from a pure *in situ* carcinoma [13]. Similar to Ca ex PA, aggressive clinical management in the early stage of SDC appears to be the only hope for good prognosis [5,6,8-11]. Thus, it is critical to establish an accurate preoperative diagnosis by fine-needle aspiration cytology, however, previous studies have indicated the difficulty of correct characterization of Ca ex PA and/or SDC due to sampling errors or inadequateness and misinterpretation [14,15].

In fact, invasive SDC ex PA could be relatively common disease, as compared with some recently published case reports of very rare tumor cell types in the salivary gland [16-18]. Despite of that, we report a case of SDC ex PA, which originated from the parotid gland and partially involved the surrounding soft tissue and lymph nodes as a rapidly increased but long-standing swelling mass. Based on the relatively inadequate cytology specimens, we preoperatively interpreted it merely as an adenocarcinoma, NOS.

## Materials and methods

The patient was an 85-year-old Japanese man. A fine-needle aspiration from the parotid gland mass was performed, followed by a radical parotidectomy. The tumor specimens after fixation in 10% neutral buffered formalin were embedded in paraffin for histological or immunohistochemical examinations. All immunohistochemical stainings were carried out using Dako Envision kit (Dako Cytomation Co., Glostrup, Denmark) according to the manufacturer’s instructions. For transmission electron microscopy (JEM-1200EX, JEOL Ltd., Tokyo, Japan), the dehydrated tissue specimens, after fixed with 2.5% glutaraldehyde and immersed in 2% osmium tetroxide, were embedded in epoxy resin.

### Case presentation

The patient had a history of benign prostatic hyperplasia 2 years ago. There was no history of malignancy, immunosuppressive disorders, use of immunosuppressive medications, or unusual infections.

He noticed a long-standing swelling mass in the right parotid gland and an enlargement of the neck lymph node 5 years before the resection. Following that, a recent rapid increase of them was presented. Laboratory data, including blood cell count, chemistry and tumor markers, were within normal limits. A neck CT scan revealed a heterogeneously enhanced and poorly-demarcated mass, measuring approximately 3 × 3 cm, in the right parotid gland. CT scans of the chest and abdomen disclosed no definite evidence of metastasis in the lymph nodes or other organs. The patient was alive and well at 8 months after the operation.

### Pathological findings

**Figure 1:**
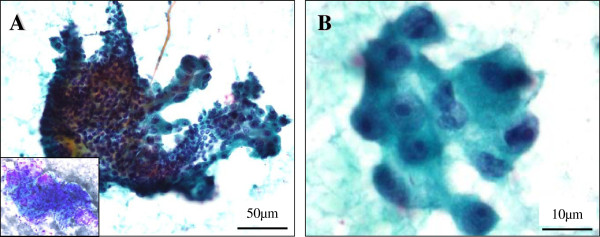
**Cytomorphologic examination of the fine needle aspiration specimens.** (**A**) The cytology specimens were inadequate, but consisted of few small clusters of cohesive and three-dimensional pleomorphic tumor cells in a papillary-like fashion (rt. side), along with flat sheets of benign monomorphic myoepithelial cells (lt. side) (Papanicolaou stains) and a small amount of metachromatic fibromyxoid stroma (inset, Giemsa stains). Bar = 50 μm. (**B**) At the periphery of these clusters and sheets, there were scattered and tiny malignant tumor cells, having relatively abundant and finely granular cytoplasm. Additionally, the nuclei were hyperchromatic, medium to large in size, pleomorphic, and often had prominent nucleoli. Bar = 10 μm.

The fine-needle aspiration cytology specimens were inadequate but consisted of few small clusters of cohesive and three-dimensional pleomorphic tumor cells in a papillary-like fashion without necrotic or hemorrhagic backgrounds (Figure 1A), along with flat sheets of benign monomorphic myoepithelial cells and a small amount of metachromatic fibromyxoid stroma, representative of benign PA (Figure 1A, inset). At the periphery of these clusters and sheets, there were few, scattered and tiny malignant tumor cells (Figure 1B). The tumor cells showed large, polygonal, and round to oval with moderate to marked pleomorphism and had relatively abundant and finely granular cytoplasm (Figure 1B). Additionally, the nuclei were hyperchromatic, medium to large in size, pleomorphic, and often had prominent nucleoli (Figure 1B). Based on that, we first interpreted it as an adenocarcinoma, NOS, and an ordinary radical parotidectomy was performed.

**Figure 2:**
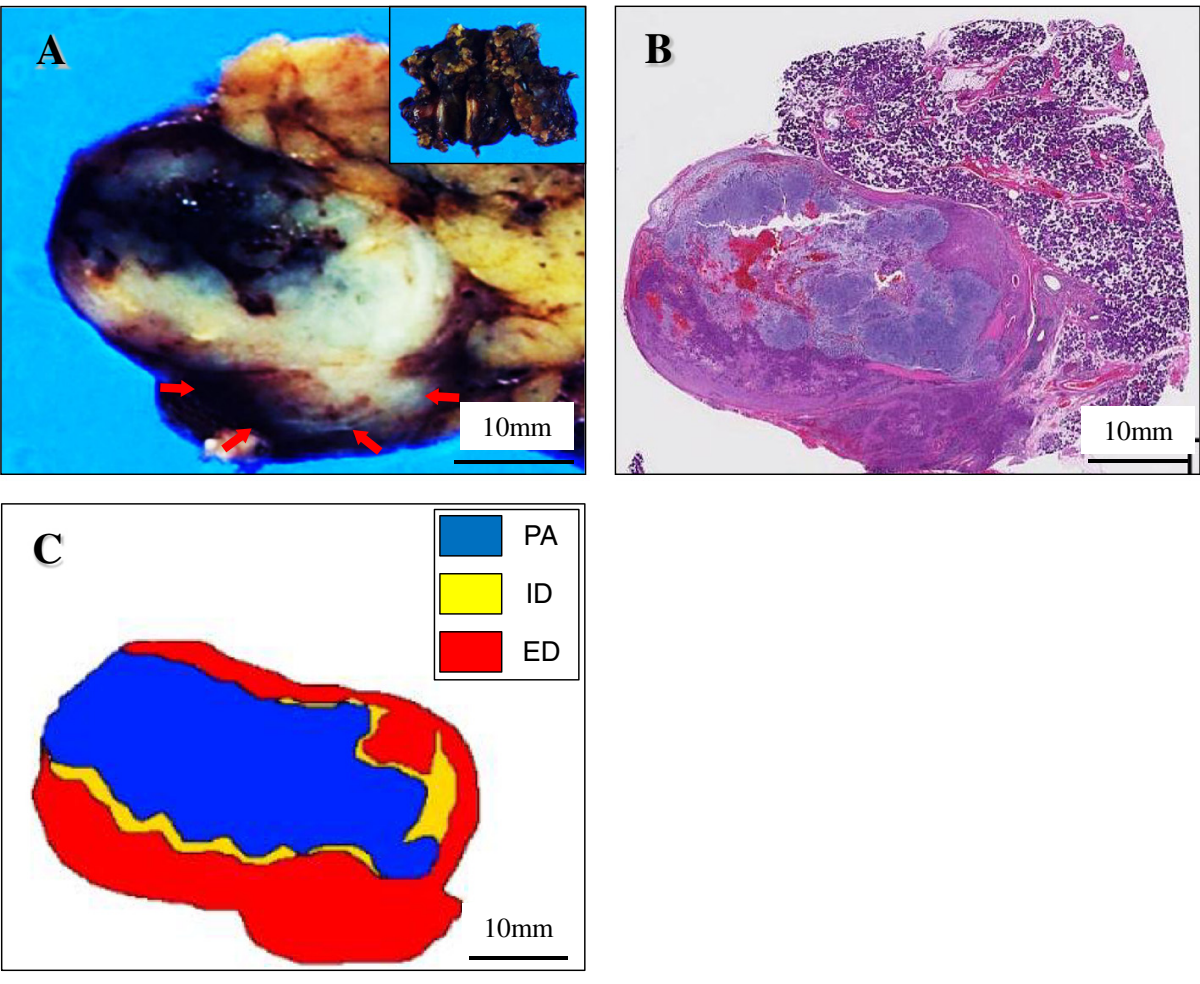
**Gross and microscopic examination, and schema of the resected specimen.** (**A**) On gross examination (inset), the cut surface revealed an encapsulated but poorly-demarcated, and predominantly solid firm mass, measuring 32 × 25 mm, which looked from grayish-blue to yellowish-white in colour, almost corresponding to the areas from pleomorphic adenoma (PA) to both ‘intraductal (ID)’ and ‘extraductal (ED)’ salivary duct carcinoma (SDC) components, associated with central hemorrhage and foci of extracapsular invasion (lower, arrows). Bar = 10 mm. (**B**) A scanning magnification of it showed that the PA components embedded in abundant chondroid matrix was less than 50%, with transition to the predominant (more than 50%) SDC components (H&E stains). Bar = 10 mm. (**C**) The schema of these tumor components (PA; blue, ID; yellow, and ED; red) was shown, likely displaying a sequential and stepwise progression.

On gross examination, the cut surface revealed an encapsulated but poorly-demarcated, and predominantly solid firm mass, measuring 32 × 25 × 12 mm, which looked from grayish-blue to yellowish-white in colour, almost corresponding to the areas from PA to SDC (Figure 2A). This tumor lesion was superimposed with central hemorrhage and foci of extracapsular invasion, replacing one part of the parotid gland (Figure 2A). A scanning magnification of it showed that the PA components embedded in abundant chondroid matrix was less than 50% in an acellular fashion, with transition to the predominant SDC components in a cellular fashion, associated with central hemorrhage (Figure 2B).

**Figure 3:**
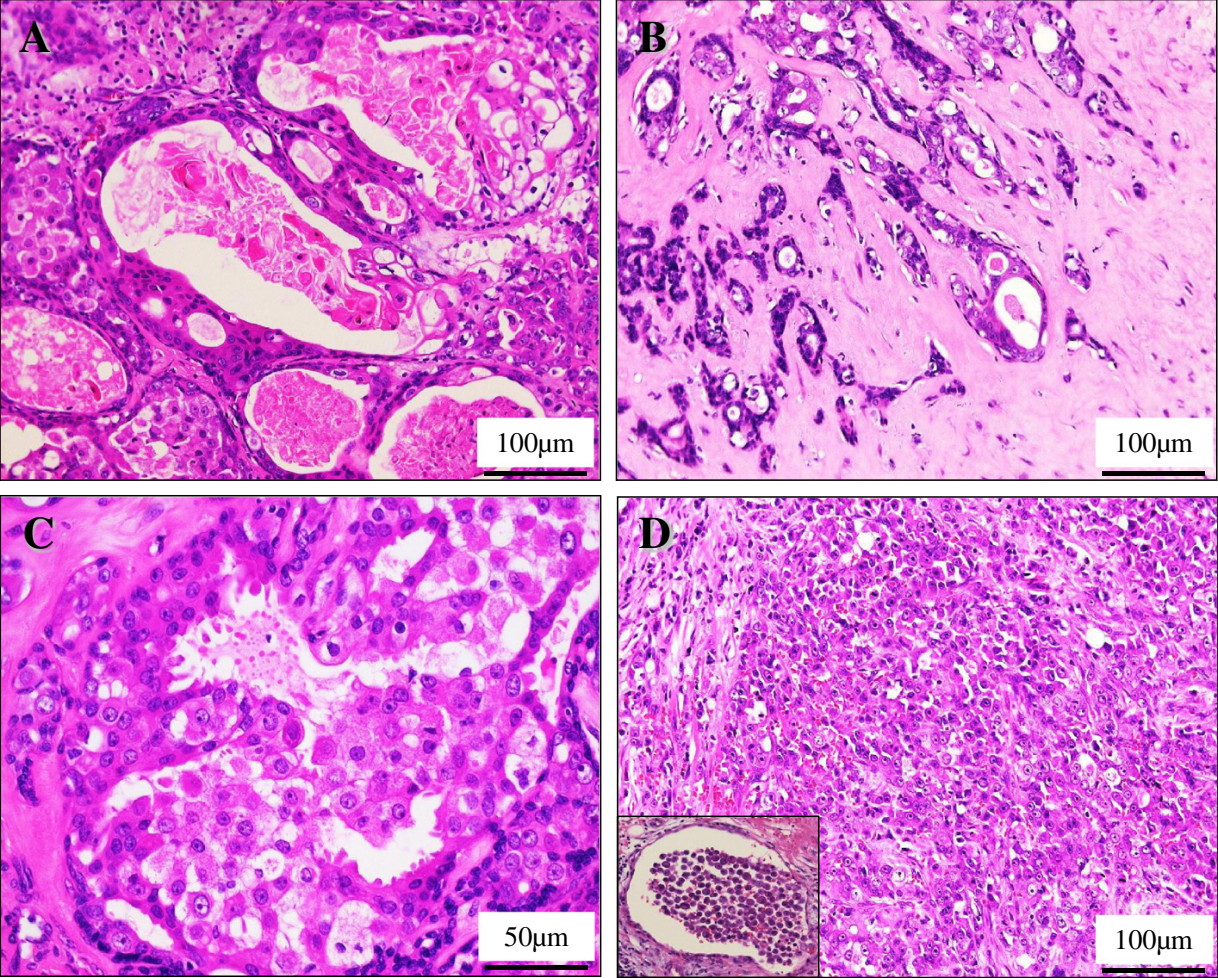
**Microscopic examination of the invasive SDC ex PA.** (**A**) In high power view, the ID components of SDC showed a proliferation of highly atypical epithelial cells having hyperchromatic pleomorphic nuclei and abundant eosinophilic cytoplasm, often arranged in a Roman-bridge appearance with foci of comedo necrosis (H&E stains, Original magnification × 200). Bar = 100 μm. (**B**) Additionally, in the PA components, the carcinoma cells occasionally displayed a minor ‘*in situ*’ component, characterized by malignant transformation of ductal luminal cells with possible preservation of the myoepithelial cell layer (H&E stains, Original magnification × 200). Bar = 100 μm. (**C**) In the ID components, the luminal adenocarcinoma cells sometimes revealed evidence of active “decapitation” secretion (H&E stains, Original magnification × 400). Bar = 50 μm. (**D**) In contrast, the ED components demonstrated an extensive infiltration to the adjacent pre-existing PA stroma and the extracapsular area, in a trabecular or alveolar fashion with severe vessel permeation (inset) (H&E stains, Original magnification × 200). Bar = 100 μm.

Microscopic findings included the presence of well-circumscribed so-called ‘intraductal (ID)’ and significantly infiltrating so-called ‘extraductal (ED)’ components of the SDC. The former ‘intraductal’ components showed a proliferation of highly atypical epithelial cells having hyperchromatic pleomorphic nuclei and abundant eosinophilic cytoplasm, often arranged in a Roman-bridge appearance with foci of comedo necrosis, typical of mammary ductal carcinoma *in situ* (Figure 3A). Although the PA components exhibited extensive chondromatous or hyalinized change, the presence of residual benign ductular structures in a two-cell pattern allowed their identification, partly in which the carcinoma cells displayed a minor ‘*in situ*’ component, characterized by malignant transformation of ductal luminal cells with possible preservation of the myoepithelial cell layer (Figure 3B). Moreover, the luminal adenocarcinoma cells sometimes revealed evidence of active “decapitation” secretion like that seen in apocrine glands (Figure 3C). In contrast, the latter ‘extraductal’ components demonstrated an extensive infiltration to the adjacent pre-existing PA stroma and the extracapsular area, in a trabecular or alveolar fashion with severe vessel permeation and many foci of lymph nodes metastases, also reminiscent of invasive ductal carcinoma of the breast (Figure 3D). These features indicated a sequential and stepwise progression very similar to breast carcinoma. The schema of these tumor components (PA; blue, ID; yellow; and ED; red) is summarized in Figure 2C.

Ultrastructural findings of the transition areas from ‘intraductal’ to ‘extraductal’ components showed that the neoplastic columnar cells occasionally with luminal microvilli had enlarged irregular nuclei with dispersed chromatin and one or more prominent nucleoli, and cytoplasm containing not only a large amount of organelles, such as endoplasmic reticulum, but secretory-like granules (data not shown). The basally-located myoepithelial cells were seen, and were neoplastic-like with enlarged irregular nuclei and cytoplasmic dense bodies of myofilaments, intermingled with collagen bands (data not shown).

**Figure 4:**
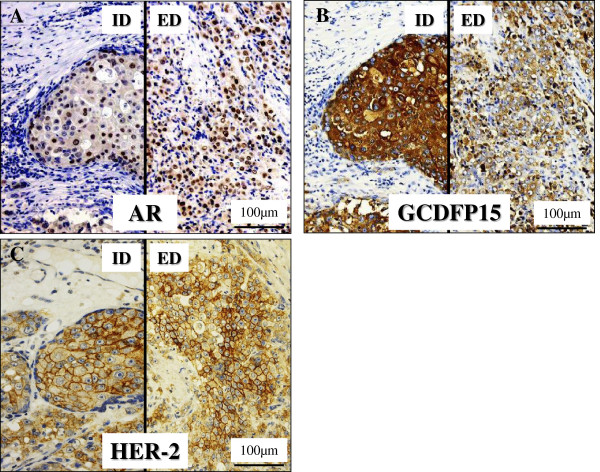
**Immunohistochemical examination of the invasive SDC ex PA.** (**A**, **B**, **C**). The carcinoma cells were strongly positive for AR (**A**), GCDFP-15 (**B**), and HER2 (**C**) in not only the ID (lt.) but ED (rt.) components. Conversely, the myoepithelial cells of the PA and ID components were negative for AR (A).

**Figure 5:**
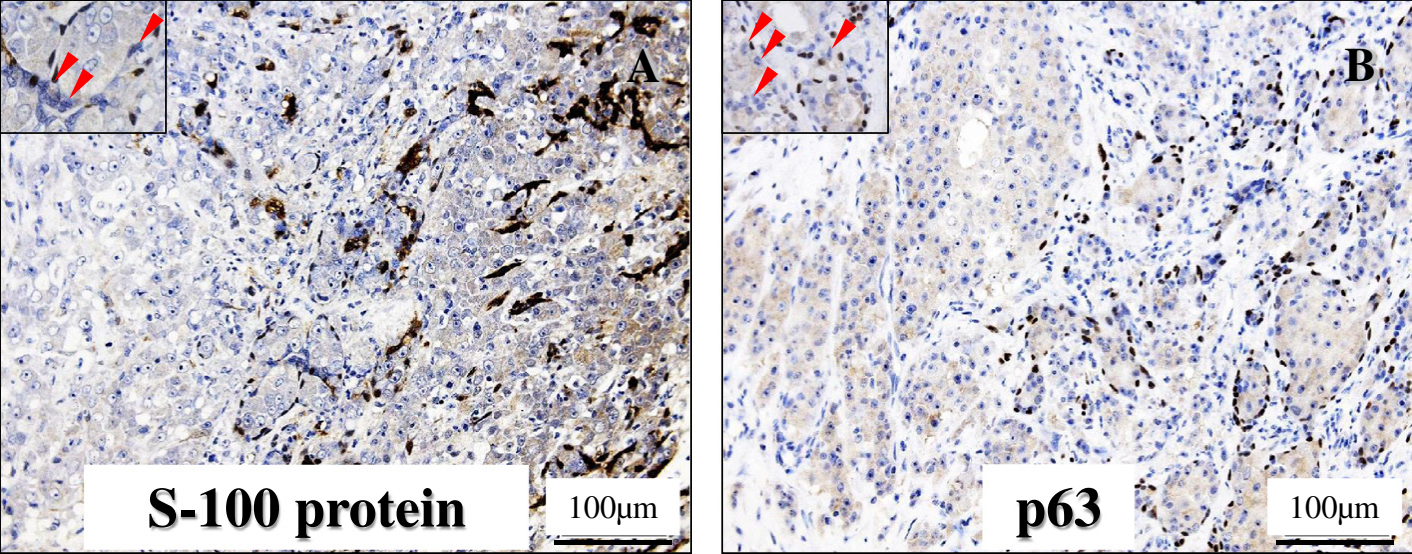
**Myoepithelial markers analysis of the invasive SDC ex PA in immunohistochemistry.** (**A**, **B**) The carcinoma cells were negative for S-100 protein (**A**) and p63 (**B**). By contrast, the myoepithelial cells of the PA and ID components were positive for S-100 protein and p63 (A-B, rt. side) (Original magnification × 200, Bar = 100 μm). However, as to the ID components, the neoplastic-like myoepithelial cells (arrowheads) having mildly enlarged and hyperchromatic nuclei were sometimes negative for both of S-100 protein (A, inset) and p63 (B, inset) (Original magnification × 400, Bar = 50 μm). Also, the lost of myoepithelial cells was focally apparent in the ID components (A-B, lt. side).

Immunohistochemically, these adenocarcinoma cells were specifically positive for androgen receptor (AR; BioGenex, San Ramon, CA, diluted 1:50) (Figure 4A) and gross cystic disease fluid protein-15 (GCDFP-15; Signet Laboratories, Inc., Dedham, MA, diluted 1:40) (Figure 4B) in not only the ‘intraductal (ID)’ but the ‘extraductal (ED)’ components, and in addition, positive for cytokeratins (AE1/AE3; CHEMICON International, Inc., Temecula, California, USA, diluted 1:200, and Cam5.2; Becton Dickinson Immunocytometry Systems, San Jose, CA, diluted 1:1), EMA (Dako, diluted 1:100), carcinoembryonic antigen (CEA; Dako, diluted 1:50), prostate specific antigen (PSA; Dako, diluted 1:40), and HER2 protein (Dako, diluted 1:1), but were negative for S-100 protein (Dako, diluted 1:900), p63 (Dako, diluted, 1:30), estrogen receptor (ER; Dako, 1:2), and progesterone receptor (PgR; Dako, 1:6) (Table 1). In the case of the HER2 protein, the results were evaluated as 3+ (intermediate to strong complete staining in > 10%) for membranous immunohistochemical expression (Figure 4C), according to the criteria of HercepTest (Dako). On the other hand, Ki67 (MIB-1; Dako, diluted 1:50) labeling index was approximately 20% or greater than 50% in the proliferating atypical cells of the ‘intraductal’ or ‘extraductal’ components, respectively, whereas less than 1% in the PA components (Table 1). Moreover, the positivity of distinct nuclear staining for p53 (Dako, diluted 1:30) was almost corresponding to the MIB-1 labeling index in each components (Table 1). In contrast, the myoepithelial cells of the PA and ‘intraductal’ components are negative for AR (BioGenex) (Figure 4A), but positive for S-100 protein (Dako) and p63 (Dako) (Figure 5A-B). However, as to the latter (‘intraductal’ components), the neoplastic-like myoepithelial cells having mildly enlarged and hyperchromatic nuclei were sometimes negative for both of them (S-100 protein; Figure 5A, inset and p63; Figure 5B, inset). Also, the lost of myoepithelial cells was focally apparent in the ‘intraductal’ components (Figure 5A-B).

**Table 1:** Immunohistochemical profile of the epithelial components in the invasive SDC ex PA

**Antibodies**	**Epithelial component**		
	**PA**	**ID**	**ED**
AR	–	+	+
GCDFP15	–	+	+
CEA	–	+	+
PSA	–	+	+
HER2	0	3+	3+
AE1/AE3	+	+	+
EMA	+	+	+
ER	–	–	–
PgR	–	–	–
S100	–	–	–
p63	–	–	–
MIBI	<1%	20%	60%
p53	<1%	20%	60%

PA: Pleomorphic adenoma, ID: “Intraductal” carcinoma, ED: “Extraductal” carcinoma.

Based on all these features, we suggested that these proliferating carcinoma cells were characteristic of apocrine differentiation, and finally made a diagnosis of invasive SDC ex PA of the parotid gland.

## Discussion

Aggressive clinical treatment in the early stage for invasive SDC is the only hope for better prognosis, due to a high grade, malignant tumor, based on the infiltrative and destructive behavior [8-11]. Thus, it is critical to establish an accurate preoperative diagnosis by fine-needle aspiration cytology, the clinical utility of which in diagnosing salivary gland tumors has been generalized. The cytologic findings of invasive SDC reflect the histopathological ones resembling invasive ductal carcinoma of the breast, showing cohesive, three-dimensional clusters and flat sheets of large and polygonal atypical cells having abundant and finely granular cytoplasm, and prominent nucleoli, arranged in an irregular branching, cribriform, or papillary growth pattern as well as single cells formation in the background of frequent necrosis [15,19]. Additionally, smears from Ca ex PA show moderate to high cellularity with pleomorphic malignant cells in the background of biphasic components of PA, displaying sheets of benign monomorphic epithelial cells along with metachromatic, fibrillary and myxoid or chondroid stroma [14]. As in the current case, although the specimens were inadequate, the cytologic features were almost similar to those as described above, even while neither necrotic backgrounds nor any cribriform formations were observed. Despite that, a confident and accurate diagnosis of SDC ex PA was impossible on cytology, suggestively due to sampling errors, lack of experience, cytomorphologic variety, and misinterpretation. Nevertheless, in cases with a strong clinical suspicion of Ca ex PA, such as ours, multiple fine needle aspiration should be performed and its suspicion should be raised to alert the pathologist, at the very least.

An immunohistochemical analysis indicates that AR and GCDFP-15 are highly and specifically expressed in more than 80% and 90% of patients with SDC, respectively, whereas ER and PgR, well known as common breast carcinoma markers, are very weakly or negatively stained [11,20-22]. Actually, one paper proposed that, when the cytologic features of high grade adenocarcinoma with a variety of cell morphology are difficult to make an accurate diagnosis, immunostaining for AR and ER on cytologic smears would be very useful for the diagnosis of SDC [19].

On the other hand, the luminal SDC carcinoma cells rarely show immunohistochemical expression of S-100 protein and p63, whereas the ‘reminiscent’ benign myoepithelial cells reveal those strong expression in the ‘intraductal’ and PA components [9-11,20-22], even while the lost of myoepithelial cells is occasionally seen in the transition areas from ‘intraductal’ to ‘extraductal’ components [20], as in our case. Indeed, myoepithelial cells can be considered as natural tumor suppressors, distinguishing between early and advanced malignant tumors in the transition from *in situ* to invasive carcinomas, and can rarely undergo malignancy. However, in the present case, the neoplastic-like enlarged myoepithelial cells in the transition areas were sometimes negative for the above myoepithelial markers. These features indicate that those cells would not have apparent myoepithelial phenotypes, but potentially neoplastic character, probably confirmed by the ultrastructural findings. We could provide the evidence for the first time that invasive SDC ex PA may arise within or from PA as a result of neoplastic transformation of outer supporting myoepithelial cells, as well as inner ductal epithelial cells. Despite of that, future studies will be further required to determine whether our suggestion is significant after collecting and examining a larger number of its cases.

## Conclusion

We herein reported a case of an invasive SDC ex PA. The present case was tentatively diagnosed as adenocarcinoma, NOS on the examination of the cytology, because its smears showed the inadequate and few components of malignant cells. All cytopathologists should be aware that its clinically and immunohistochemically characteristic features, as well as multiple and adequate fine needle aspiration, could lead to a correct diagnosis.

## Consent

Written informed consent was obtained from the patient for publication of this case report and any accompanying images. A copy of the written consent is available for review by the Editor-in-Chief of this journal.

## Competing interests

The authors declare that they have no competing interests.

## Authors’ contributions

SY and AN participated in conception of the idea and writing of the manuscript. SY, AN, TT, XG, TT, KYW, SS and YS performed the cytohistological and ultrastructural interpretation of the tumor tissue. All authors have read and approved the final manuscript.
